# 3D-FVS: construction and application of three-dimensional fundus vascular structure model based on single image features

**DOI:** 10.1038/s41433-022-02364-0

**Published:** 2022-12-15

**Authors:** Zhaomin Yao, Renli Luo, Chencong Xing, Fei Li, Gancheng Zhu, Zhiguo Wang, Guoxu Zhang

**Affiliations:** 1 College of Medicine and Biological Information Engineering, Northeastern University, Shenyang, Liaoning 110167 China; 2 Department of Nuclear Medicine, General Hospital of Northern Theater Command, Shenyang, Liaoning 110016 China; 3 School of Computer Science and Software Engineering, East China Normal University, Shanghai, 200241 China; 4 College of Computer Science and Technology, and Key Laboratory of Symbolic Computation and Knowledge Engineering of Ministry of Education, Jilin University, Changchun, Jilin 130012 China

**Keywords:** Eye manifestations, Predictive markers

## Abstract

**Background:**

Fundus microvasculature may be visually observed by ophthalmoscope and has been widely used in clinical practice. Due to the limitations of available equipment and technology, most studies only utilized the two-dimensional planar features of the fundus microvasculature.

**Methods:**

This study proposed a novel method for establishing the three-dimensional fundus vascular structure model and generating hemodynamic characteristics based on a single image. Firstly, the fundus vascular are segmented through our proposed network framework. Then, the length and width of vascular segments and the relationship among the adjacent segments are collected to construct the three-dimensional vascular structure model. Finally, the hemodynamic model is generated based on the vascular structure model, and highly correlated hemodynamic features are selected to diagnose the ophthalmic diseases.

**Results:**

In fundus vascular segmentation, the proposed network framework obtained 98.63% and 97.52% on Area Under Curve (AUC) and accuracy respectively. In diagnosis, the high correlation features extracted based on the proposed method achieved 95% on accuracy.

**Conclusions:**

This study demonstrated that hemodynamic features filtered by relevance were essential for diagnosing retinal diseases. Additionally, the method proposed also outperformed the existing models on the levels of retina vessel segmentation. In conclusion, the proposed method may represent a novel way to diagnose retinal related diseases, which can analysis two-dimensional fundus pictures by extracting heterogeneous three-dimensional features.

## Introduction

Various ophthalmic and systemic diseases can cause varying degrees of deformation on fundus microvasculature. Observing the morphological changes of optic disc vessels is one of the informative diagnostic factors of glaucoma [1, 2]. The progress of diabetic angiopathy can be assessed by the degree of deformation of the fundus vessels [3–5]. This sign may also occur in patients with certain types of hypertensions [6–9]. Additionally, people with deformed fundus vessels have a higher risk of stroke than normal people [10].

Most studies investigated the fundus microvasculature-based disease prediction problems by only the two-dimensional planar features, both on supervised and unsupervised methods [11–13]. The proposed method defined the ratio of the blood vessels area in the inferior-superior side to the area of blood vessels in the nasal-temporal side as a feature and optimized a glaucoma prediction model with 100% in sensitivity and 80% in specificity [14]. A prediction model of hypertensive retinopathy was proposed using the features of artery vein ratios(AVR), diameter and tortuosity of blood vascular and achieved 92% in accuracy [15]. Lacunar stroke also demonstrated significant changes in the fundus microvasculature and may be detected using the features of retinal fractal dimension, which is a quantitative measure of microvascular branching complexity and density [16].

To the best of our understanding, there is no research on the three-dimensional structure of vascular based on single fundus image at present. Thus, this study proposed a novel method, which can build fundus microvasculature model by mining and integrating vascular related spatial information contained in a single image. Firstly, the fundus microvascular segmentation map is obtained by our proposed DenseBlock-Unet framework. Then, the skeleton image with central points and the vessel boundaries image can be obtained from the previous segmentation map through the Zhang Suen thinning algorithm and canny edge detection algorithm respectively. After that, relevant spatial information will be extracted and integrated into the software the SimVascular (SV) [17] to generate a three-dimensional model and complete the hemodynamic model simulation. Finally, the feature information generated by the model is used to diagnose whether the target image has ophthalmic diseases.

## Materials and methods

### Dataset

The publicly available datasets STARE [18, 19] was used to test our proposed algorithm. This dataset contained 20 images with a size of 605×705 pixels. Two manually labelled vessel ground-truth were provided and the first one was usually used as the gold standard. Because the author didn’t separate the training and testing dataset from these images, we evaluated the performance by performing leave-one-out cross-validation at the stage of retina vessel segmentation as other works had been done. In addition, the author labelled all these images, thus the proposed classification model can be tested base on the gold standard.

### Retina vessel segmentation

**Fig. 1:**
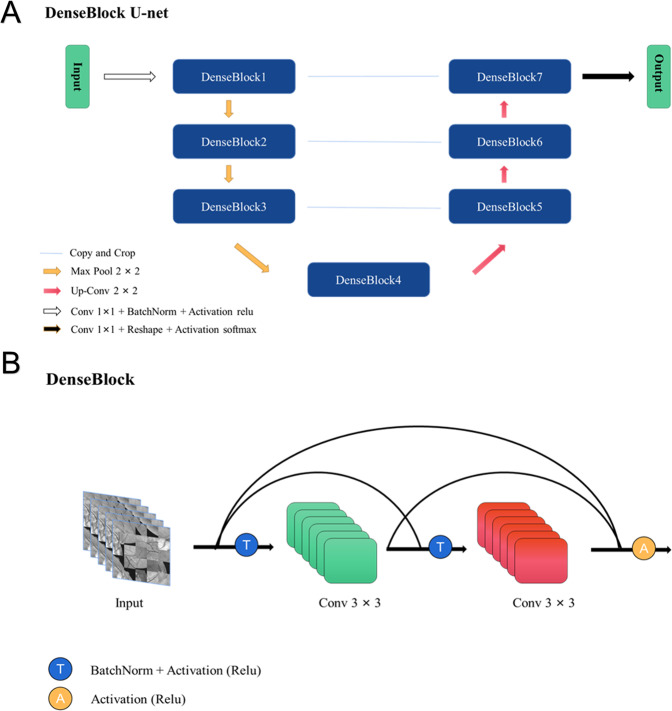
The Details of the DenseBlock-Unet. **a** The framework of DenseBlock-Unet. **b** The DenseBlock in DensenBlock-Unet.

Inspired by Unet [20], A novel method named The DenseBlock-Unet was proposed for Image Segmentation in Bio-medical. The architecture of The DenseBlock-Unet contains two paths. The first one is the contraction path which is used to capture the context in the image. The second one is the symmetric expanding path which is used to enable precise localization using transposed convolutions. The DenseBlock [21] in the DenseBlock-Unet contains two convolution layers for reducing artifacts from images and enabling the neural network to be more deeper. Therefore, its performance in segmentation task can be better than primary Unet. The architecture of the DenseBlock-Unet is shown in Fig. 1a.

DenseBlock is really helped to remove artifacts from images. The neural networks using DenseBlock layers can be deeper than classical networks composed by convolution layers, which means useful information can be extracted immensely. What’s more, its skip connection not only connects the upper and lower layers, but also contains cross layer connection directly. The gradient obtained by every layer is the gradient addition from the previous layers. Which is showed at great length in Fig. 1b.

As Fig.1b shows that DenseBlock contains two convolution layers and its input is last DenseBlock after max pooling by 2×2. A composite function of two consecutive operations: batch normalization, followed by a rectified linear unit is used for convolution layers’ connection.

Most of the existing models only using the G channel for the segmentation task [22–24]. However, considering the R channel may contain some important features, the proposed method firstly uses three different proportions of channels which are 0.5G + 0.5R, 0.75G + 0.25R, and 1G + 0R for segmentation tasks. Then the proposed method performs the Local Search Algorithm around the best proportion gotten from the above experiments to find the best local maximum value. The proportion in the Local Searching Algorithm is expressed as *(g* + i*step)G + (r-i*step)R where the step is 0.01 and the g and r are the best proportion getting from the above experiments.

### Vascular structure surface model

Considering the optic disc was one of the widest research areas in ophthalmic related diseases, our method only studied on it and these areas were labelled by a professional doctor firstly. In this stage, the methods used to build vascular structure surface models were the same as which was implemented in SimVascular, a software used to construct hemodynamic model in the following steps. Firstly, this method required to extract the centreline and downsample the points on it. Then the radius of cross-section corresponding to the point will be calculated and circles with these points as the centre will be drawn. Finally, these contours will be connected smoothly to form the vascular structure surface model.

**Fig. 2:**
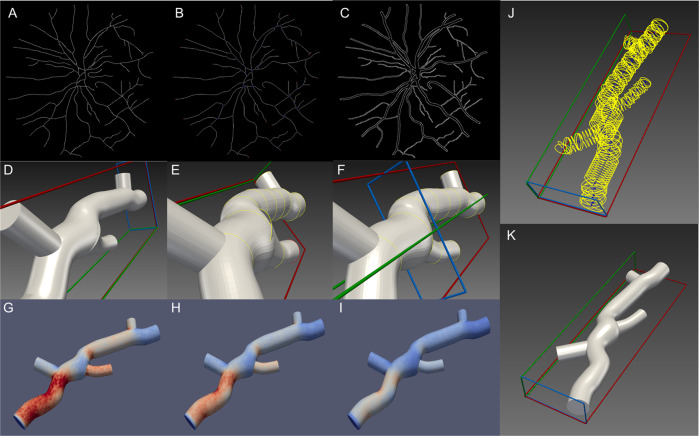
The modelling process and results. **a** Vascular skeleton line. **b** Endpoints and intersections on the skeleton line. **c** Vascular boundary. **d** The model highlights a part. **e** Model protruding silhouette. **f** After adjusting the highlighted outline. **g** Pressure distribution just after blood flow into blood vessels. **h** Pressure distribution where most of the blood flow has entered the blood vessel. **i** Vascular pressure distribution at the end of blood flow. **j** Too many contours on the skeleton line cause crossover. **k** Sample optic disc area vascular model.

This study utilized the Zhang-Suen thinning algorithm to skeletonize the segmented fundus vascular images. Each iteration step of this algorithm was to corrode the target pixels satisfying certain conditions. This algorithm was iteratively executed until no new pixels were corroded. The output of this skeletonization step was illustrated in Supplementary Fig. 1a. Then all the points on the skeleton line will be traversed for finding the coordinates of the endpoints and intersections of the skeleton line, as shown in Supplementary Fig. 1b, by calculating the number of points near the point with the size of 3 × 3 area. When the points on the skeleton line were too dense, it will lead to the intersection between the corresponding contours of every two points (which was shown in Fig. 2j), and the blood vessel modelling process cannot be completed at this time. Therefore, downsample was used to prevent intersections between the corresponding contours of every two feature points by removing points in a fixed number of intervals.

The canny operator was utilized to detect the edges in the fundus images, as shown in Supplementary Fig. 1c. The canny operator may not only improve the sensitivity of detecting the vessel edges, but also suppress the image noises. For each point selected on the centreline, we use the normal vector direction of the vessel segment where the point is located to determine the shortest distance (Euclidean distance) from the edge of the vessel on the edge map (Fig. 2c). We consider this distance to be the radius of the blood vessel profile at which the point is located. Through the above operations, the centreline and radii of the retinal vascular network are obtained.

When generating complex 3D models, more problems are prone to occur. As shown in Fig. 2e, the gaps at the intersection of the blood vessels occur because the difference in radius between the two blood vessels at the intersection is too large. The appearance of gaps between blood vessels does not meet the actual situation, and the model needs to be modified to meet the hemodynamic simulation. The gaps at the intersection of blood vessels can be resolved by adjusting the radius of the protruding contour. SV provides multiple methods for model modification, and users can adjust the model according to their needs. Here we first adjusted the radius of the protruding contour, then eliminated the gaps at the intersection of the blood vessels based on the local Laplacian smoothing method [25], making the model surface smoother. The sample final model was shown in Fig. 2k.

### Hemodynamic modelling

We follow the requirements [26–28] of the software to set different parameters to describe boundary condition of inlets and outlets and obtain three kinds of features which were flowrate for each face with time steps, pressure for each face with time steps, the average, maximum, minimum values of pressure, flowrate for each face. To sum up, there are 140 features for each sample.

## Results and discussion

### Evaluation of retina vessel segmentation

In the same way as most of the existing methods, the proposed method regards the retinal microvascular segmentation result as a binary classification problem which is composed of vessel pixels(positive) and other pixels(negative). After comparing with the manual ground truth, four measures can be calculated, i.e., true positives (TP), false negatives (FN), true negatives (TN), and false positives (FP). Four performance metrics are used to illustrate the good ability of the model for vascular segmentation, i.e., accuracy (ACC), sensitivity (SE), specificity (SP) and Area Under the ROC curve (AUC). The performance metrics of the proposed method on STARE dataset with three kinds of proportion are shown in Table 1.

**Table 1:** performance of three kind of proportion.

G	R	AUC(%)	ACC(%)	SE(%)	SP(%)
0.5	0.5	98.09	97.04	80.11	98.43
0.75	0.25	98.42	97.41	81.69	98.7
1	0	98.63	97.52	81.8	98.82

The proportion of 1G + 0R obtains the best performance. After performing the Local Search Algorithm, the performance of proportion around 1G + 0R is declining, which means the 1G + 0R is the local best proportion. The AUC result at the proportion of 0.99G + 0.01R is 98.18%. The result of extracted blood vessel images was shown in Supplementary Fig. 2.

As shown in Table 2, the proposed method obtained best accuracy among all the methods [23, 29–35] we know, which was 97.52% and at least 0.57% higher than others. Additionally, other indicators were also relatively excellent, especially specificity was also the highest.

**Table 2:** Evaluation of retina vessel segmentation.

Methods	AUC (%)	ACC (%)	SE (%)	SP (%)
Lam et al. [29]	97.39	95.67	NA	NA
Fraz et al. [30]	NA	94.42	73.11	96.8
Fraz et al. [31]	97.68	95.34	75.48	97.63
Li et al. [32]	98.79	96.28	77.26	98.44
Azzopardi et al. [33]	94.97	95.63	77.16	97.01
Mapayi et al. [34]	NA	95.10	76.26	96.57
Liskowski et al. [23]	99.30	96.67	92.89	97.10
Tang et al. [35]	98.98	96.95	81.62	98.69
Our method with best local optimal proportion	98.63	97.52	81.8	98.82

### Evaluation of vascular structure surface model

Here we used grid quality analysis to evaluate our vascular structure surface model. At the same time, we also compared it with two famous methods based on blood vessel centreline, which were atmull-Clark Surface Method (CCS) and Adaptive Loop Surface Method (ALS).

The assembly meshing method of the software ANSYS was used to divide the blood vessel model into triangular meshes and to analyse the quality of these meshes. Additionally, four indicators, which were element quality, aspect ratio, maximum internal angle, and skewness, were applied to perform the mesh quality analysis. Element quality is the ratio of the volume to the side length of a given element. Its value is between 0 and 1. The worst is 0 and the best is 1. Aspect ratio represents the ratio of the radius of the inscribed circle to the radius of the inscribed circle, the best value is 1, and the bigger the worse. The maximum internal angle evaluates the shape of triangular mesh. The closer the value is to 60 degrees, the better. And Skew rate is the difference between the actual node shape and the equivalent volume node, the value between 0 and 1, the closer the value is to 0, the better.

Table 3 lists the average of 20 models generated by the three methods under the five indicators. It can be seen that the SV method is better in terms of element quality and range of skewness. The aspect ratio is only 0.01 worse than CSS, and in terms of maximum internal angle, it is basically the same as the CSS and ALS methods. The CSS and ALS methods can adjust the intersection of models, resulting in less stable mesh quality in the model. As the model is adjusted, the mesh changes in the model are smaller and consequently the maximum internal angle is smaller. Generally, because the SV method does not adjust the model intersection, the quality of the generated model mesh is more stable and closer to the actual situation features correlation analysis in our project.

**Table 3:** Comparison results of three modelling methods.

Methods	Element quality	Aspect ratio	Maximum corner angle	Skewness
SV	0.8278 (±0.0016)	1.880 (±0.0057)	96.85° (±0.0024)	0.2432 (±0.0027)
CCS	0.8274 (±0.0027)	1.879 (±0.0100)	96.75° (±0.0045)	0.2441 (±0.0045)
ALS	0.8262(±0.0048)	1.884 (±0.0168)	96.82° (±0.0034)	0.2460 (±0.0075)

### Evaluation of features

By way of better fitting performance and reducing over-fitting, some features were selected for classification tasks. The feature selection procedure was made up of three parts. Firstly, Anova (T-test) algorithm extracted features which p-values less than 0.05 in traditional statistical inference. The result showed that there were 88 features selected by the algorithm respectively, and the best classification performance was 85% by Logistic Regression classifier. Secondly, Anova (F-test) algorithm with retaining features less than the threshold obtained by certain step length iteration. The result showed that 4 features were selected by this method, and the best classification performance was 95% by the SVM classifier. Thirdly, the performance of the best classification using the best first search algorithm on the features was 85% by SVM classifier. Additionally, there were 39 features in this procedure and four of them performed best, which were “Qmax_Time”, “Qmax_Time_from_cm”, “Step_10_mean”, and “Step_12_mean”. The “Qmax_Time” feature means the time when the mean flowrate inside the vessel reaches a maximum. When the flow rate is L/min, the function of “Qmax_Time_from_cm” is the same as that of “Qmax_Time”. while the flowrate is L/min. The Step_10_mean and the Step_12_mean features are the mean flowrate at the time of the 10th step and the 12th step. Coincidentally, two features both mean max time and another two features both are the means of steps. The result of the classification implied that the first three output files can be used for retinal image classification.

### Evaluation of classification

Based on the above features of great relevance, some commonly used classifiers with better effect i.e., Extreme Gradient Boosting (XGBOOST), Support Vector Machine (SVM), Nearest Neighbor (NN), Decision Tree (DTree), Naive Bayes (NBayes), Logistic Regression (LR), and Random Forest (RF) were used. Among them, the effect on Support Vector Machine (SVM) was the best, reaching 95%. At the same time, two different types of characteristics (HOG and LCP) were also applied to test these 20 original images for comparison. Here we used the same feature selection methods and classifiers as above. In addition, all possible parameters for the two kinds of algorithms were set with different experiments. Finally, the mean best accuracy of HOG and LCP was 80% and 87% respectively. What’s more, there was only one result in LCP and none in HOG better than the proposed method in all experiments.

### The display of hemodynamic model

We made a video of the hemodynamic simulation results of 20 models, and selected three screenshots from the hemodynamic simulation results of picture 235 of the STARE dataset for demonstration purposes. In the hemodynamic simulation results, the darker the red, the greater the pressure, and the darker the blue, the less the pressure. Figure 2g shows the situation when the blood flow has just entered the blood vessel. The blood flow pressure at the entrance is the largest, and certain pressure in the rest of the blood vessel. Figure 2h shows that most blood flow has entered the blood vessel, and the pressure at the blood vessel entrance is reduced. Figure 2i shows that the blood flow inflow process has ended, so the pressure in each part of the blood vessel becomes smaller. All hemodynamic simulation process videos can be found in Supplementary File 1.

### The advantages of hemodynamic model

It is common for patients undergoing ophthalmic surgery to suffer from microcirculation disorders, insufficient fundus blood supply, and hemodynamic abnormalities [36, 37]. The factors listed above lead to an increase in blood viscosity, microthrombosis, slow blood flow, capillary occlusion, as well as hypoxia and ischemia of the fundus tissues [38, 39]. Almost all of these signs can be intuitively expressed by hemodynamic models. At the same time, this convenient non-invasive technology is capable of detecting patients on a regular basis. As soon as a patient is detected to have complications following surgery, drugs can be administered immediately. It is anticipated that these drugs will promote the formation of fundus collateral circulation, improve the microcirculation of the fundus, and increase the supply of blood to the fundus. Consequently, vision can be improved significantly and lesions in the fundus can be reduced in extent [40].

### Relationship between image resolution and machine learning systems

The training of a machine learning system generally requires a sufficient number of low-level and high-level input features. Low-level features are typically small details in an image, such as edges, corners, colours, pixels, etc. The high-level features are derived from low-level features and are useful for specifying an object’s shape with a greater amount of semantic information [41]. Accordingly, the higher the resolution of the images, the better the performance of the machine learning system. However, it is difficult to obtain due to equipment limitations. This study utilized the best colour channel ratios to provide additional information to the model. Other methods, such as transfer learning and super-resolution image processing, can also enhance machine learning performance.

Supplementary information is available at nature.com/eye

### Summary

#### What was known before

- Fundus microvascular structure A. Fundus microvasculature may be visually observed by ophthalmoscope and has been widely used in clinical practice B. Various ophthalmic diseases and heart cerebrovascular disease can cause varying degrees of deformation on fundus microvasculature
- Hemodynamic model Haemodynamics is a branch of biomechanics. Its main task is to apply the theories and methods of fluid mechanics to study the causes, conditions, states and various influencing factors of blood circulation along blood vessels, so as to clarify the law of blood flow, physiological significance and the relationship with diseases

#### What this study adds

- Construction of Three-Dimensional Fundus Vascular Structure Model Most of the previous studies were based on the plane features of fundus images. However, our research has made a breakthrough. By extracting and integrating the spatial features of a single fundus image, we have carried out three-dimensional vascular model.
- Diagnose related diseases through the hemodynamic characteristics of fundus. Haemodynamics has been widely used in the diagnosis of diseases, but due to the particularity of ophthalmic vessels, there has been no relevant research. Therefore, we have made a preliminary attempt.

## Supplementary information

Supplementary Fig. 1

Supplementary Fig. 2

Supplementary File 1

Supplementary Information

## Data Availability

The datasets generated during and/or analysed during the current study are available in the STARE repository, https://cecas.clemson.edu/~ahoover/stare/.
