# Supplementary figures and images for: 3D-FVS: construction and application of three-dimensional fundus vascular structure model based on single image features
Source: Eye (Lond). 2022 Dec 15;37(12):2505–10. doi: 10.1038/s41433-022-02364-0 (PMC10397231; doi:10.1038/s41433-022-02364-0)

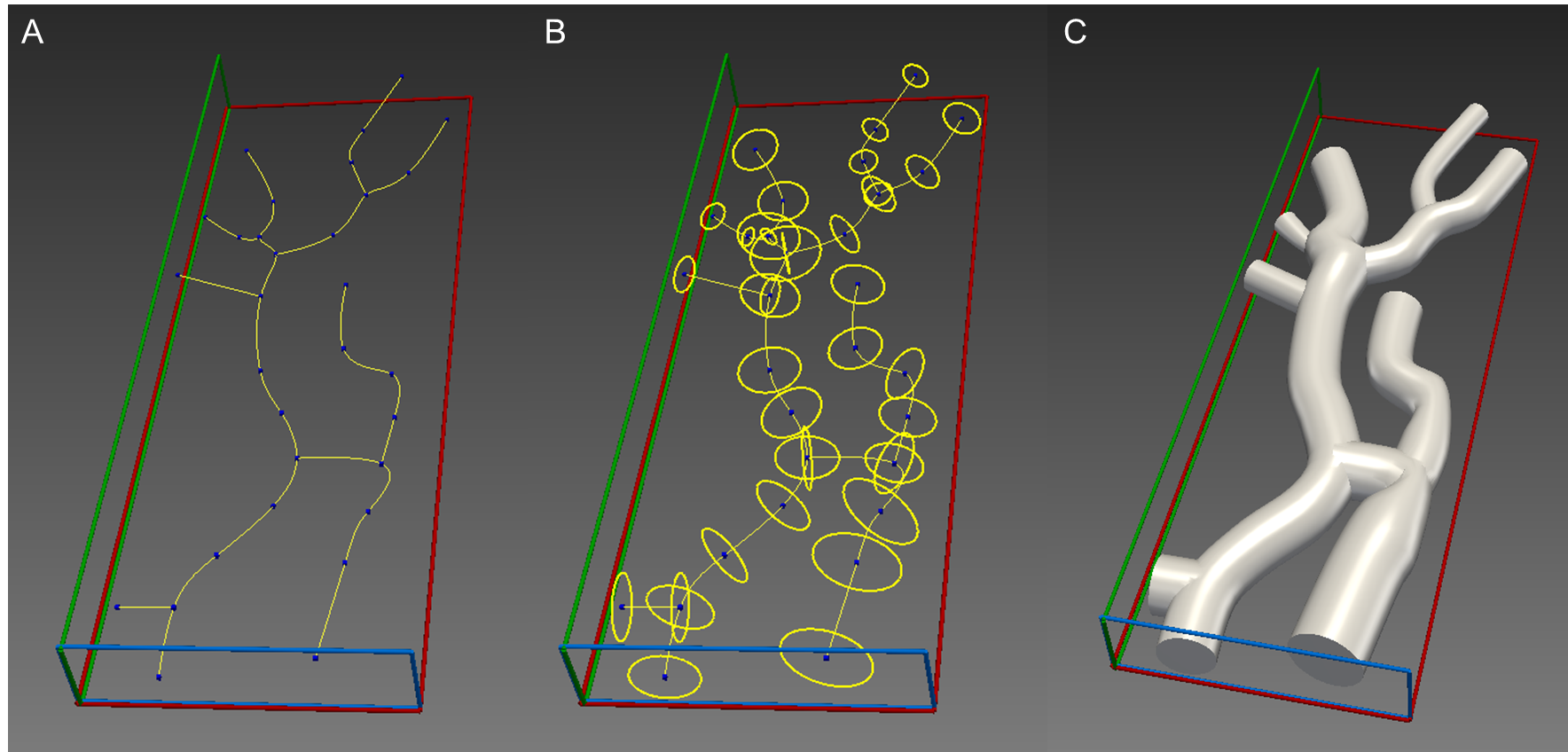

Supplement: Supplementary file 1 — Supplementary Fig. 1 [file 41433_2022_2364_MOESM1_ESM.tif]

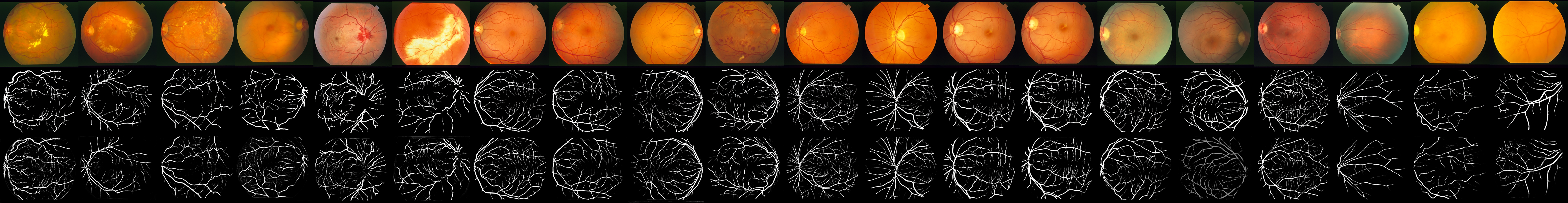

Supplement: Supplementary file 2 — Supplementary Fig. 2 [file 41433_2022_2364_MOESM2_ESM.tif]
