# Supplementary material for: 3D-FVS: construction and application of three-dimensional fundus vascular structure model based on single image features
Source: Eye (Lond). 2022 Dec 15;37(12):2505–10. doi: 10.1038/s41433-022-02364-0 (PMC10397231; doi:10.1038/s41433-022-02364-0)
Supplement: Supplementary file 4 — Supplementary Information [file 41433_2022_2364_MOESM4_ESM.docx]

# Supplementary Information

Supplementary Figure 1. Modelling process of vascular structure surface (a) Centre line and down sample point (B) The radius of cross section (C) The vascular model

Supplementary Figure 2: The segmentation result for the STARE dataset. The first row through the third row are retinal images, manual segmentation and the segmentation result of the proposed method.

Supplementary File 1: All hemodynamic simulation process videos.
